# Intermittent Fasting for Twelve Weeks Leads to Increases in Fat Mass and Hyperinsulinemia in Young Female Wistar Rats

**DOI:** 10.3390/nu12041029

**Published:** 2020-04-09

**Authors:** Ana Cláudia Munhoz, Eloisa Aparecida Vilas-Boas, Ana Carolina Panveloski-Costa, Jaqueline Santos Moreira Leite, Camila Ferraz Lucena, Patrícia Riva, Henriette Emilio, Angelo R. Carpinelli

**Affiliations:** 1 Department of Physiology and Biophysics, Institute of Biomedical Sciences, University of Sao Paulo, 1524 Professor Lineu Prestes avenue, Butanta, São Paulo 05508-900, Brazil; elovilasboas@usp.br (E.A.V.-B.); anakpan@gmail.com (A.C.P.-C.); jaqueline.leite@usp.br (J.S.M.L.); cflucena07@gmail.com (C.F.L.); patricia.riva@gmail.com (P.R.); angelo@icb.usp.br (A.R.C.); 2 Department of General Biology, Ponta Grossa State University, 4748 General Carlos Cavalcanti avenue, Uvaranas, Parana, PR 84030-900, Brazil; henry.emilio@gmail.com

**Keywords:** intermittent fasting, fat mass, insulin secretion, pancreatic islet

## Abstract

Fasting is known to cause physiological changes in the endocrine pancreas, including decreased insulin secretion and increased reactive oxygen species (ROS) production. However, there is no consensus about the long-term effects of intermittent fasting (IF), which can involve up to 24 hours of fasting interspersed with normal feeding days. In the present study, we analyzed the effects of alternate-day IF for 12 weeks in a developing and healthy organism. Female 30-day-old Wistar rats were randomly divided into two groups: control, with free access to standard rodent chow; and IF, subjected to 24-hour fasts intercalated with 24-hours of free access to the same chow. Alternate-day IF decreased weight gain and food intake. Surprisingly, IF also elevated plasma insulin concentrations, both at baseline and after glucose administration collected during oGTT. After 12 weeks of dietary intervention, pancreatic islets displayed increased ROS production and apoptosis. Despite their lower body weight, IF animals had increased fat reserves and decreased muscle mass. Taken together, these findings suggest that alternate-day IF promote β -cell dysfunction, especially in developing animals. More long-term research is necessary to define the best IF protocol to reduce side effects.

## 1. Introduction 

The increasing prevalence of obesity around the globe is known to be linked to unhealthy eating patterns and a sedentary lifestyle. Treatments in obesity involve hypocaloric diets associated with physical exercises, producing an overall energy deficit [1,2] and leading to weight loss. One of the many diets that results in weight loss in both humans and laboratory animal models is intermittent fasting (IF). The most common IF protocols adopted by people in attempt to lose weight involve daily fasting for up to 16 hours; or fasting periods of up to 24 hours interspersed with normal feeding days [3,4]. Despite demonstrated weight loss [5,6,7], more studies are needed to evaluate whether alternate-day IF diets promote health benefits or could cause undesired effects in the long run. 

Several studies about IF in both volunteers and animal models have uncovered beneficial effects, such as improved insulin sensitivity and glucose homeostasis [8,9,10]; improved performance and metabolic efficiency during exercise [11], increased alertness [12], and increased life expectancy [13,14,15]; a reduction in blood pressure and heart rate [16,17,18,19,20]; a reduction in inflammation; and protection against neurodegeneration [17,18,19,21,22,23,24,25,26,27,28,29,30]. 

On the other hand, many studies have reported adverse outcomes as a consequence of IF. Diabetic individuals performing IF may exhibit hypoglycaemia, ketoacidosis, dehydration, hypotension, and thrombosis [10,31]. A study in middle-aged men showed a significant increase in blood pressure and total cholesterol [32]. Increased plasma concentrations of cortisol at night have also been reported in several studies following daily fasting for one month, suggesting altered circadian rhythms [33,34,35,36,37,38,39,40]. IF may lead to the worsening of glucose tolerance in non-obese women [41] and cause decreased energy expenditure in young women [42]. Munsters et al. compared plasma insulin and glucose concentrations over only three days of reduced meal frequency and found that the intermittent fasting model produced higher peaks and more abrupt declines in insulin and glucose concentrations, indicating a biological environment prepared for long-term insulin resistance and diabetes [43]. 

Various physiological parameters are known to be altered in the endocrine pancreas during acute fasting, including insulin syntheses, glucose-stimulated insulin secretion (GSIS), glucose utilization, pancreatic islet metabolism, and pancreatic β-cell sensitivity to glucose [44,45,46,47,48,49,50]. Our group showed that a 48-hour acute fast increases net reactive oxygen species (ROS) production in isolated pancreatic islets and alters GSIS [51]. Thus, in the present study, we sought to evaluate the effects of 12 weeks of IF on glucose homeostasis and pancreatic islets isolated from rats.

## 2. Material and Methods

### 2.1. Ethical Approval

The Ethical Committee on Animal Research of the Institute of Biomedical Sciences of the University of São Paulo (CEUA) and Brazilian Society of Science in Laboratory Animals (SBCAL) approved the experimental protocols for this study, including the use of 24-hour fasted rats. The approved protocol number is 157/2014/CEUA.

### 2.2. Animals

**Figure 1:**
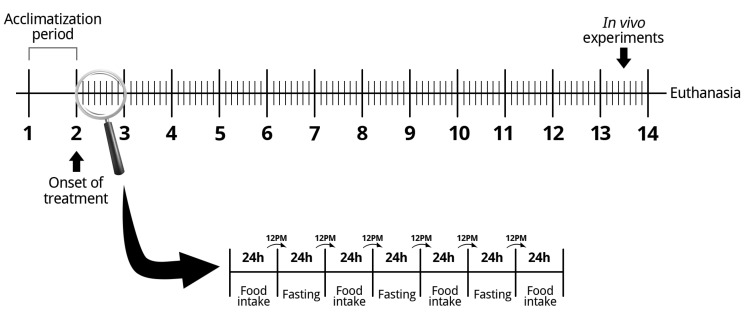
Scheme of the intermittent fasting protocol used.

Three-week-old female Wistar rats were transferred from the breeding facility to the experimental facility and remained 1 week for acclimatization before IF is initiated. The animals were housed in cages with three animals each in a room with constant temperature of 23 ± 2 °C on a standard 12-hour light/dark cycle. After acclimatization, the animals were randomized into two groups: control (CT) and intermittent fasting (IF) for 12 weeks. During the treatment, the CT group had free access to the standard rodent chow (Nuvilab, Sao Paulo, SP, Brazil – macro-nutrients in supplemental Figure S1) and the IF group was submitted to periods of 24 hours of total food deprivation interspersed with periods of 24 hours of ad libitum access to the same standard rodent chow. Food was withdrawn or made available to the IF group at noon (Figure 1). Both groups had free access to water for the entire period. During the full period, body weight and food intake were recorded. During the last week of treatment, we performed an oral glucose tolerance test (oGTT), an intraperitoneal insulin tolerance test (iITT), and dual x-ray absorptiometry. After 12 weeks of treatment, the animals were killed, and the pancreas, blood, liver, adipose tissue, and muscle were collected for different analyses. All tissue weights collected were normalized by the body weight of the animals. Euthanasia was always performed after the 24-hour period of free access to food, so that both groups were fed, thus eliminating the need to subject the control animals to acute fasting and excluding its effects. The exception occurred in liver collection, in order to analyze glycogen content after acute fasting, so, in this way, liver collection was also performed after a 24-hour fast of both groups in two different cohorts (i.e., both groups with free access to food or both groups in 24-hour fasting). 

### 2.3. Indexes Calculation 

The Lee index was calculated using the body weight (grams) and naso-anal length (centimeters) of the animals. The Lee index and adipose tissue mass correlate and can be used as a simple measure of obesity in rats [52].

(1) $$Lee\;index=\frac{\sqrt[{3}]{\mathrm{body}\;\mathrm{weight}}}{\mathrm{naso}\_\mathrm{anal}\;\mathrm{length}}$$

The Homeostasis Model Assessment (HOMA) index is based on fasting plasma insulin (ng/ml) and glucose concentrations (mg/dL) [53]. Its purpose is to determine insulin resistance (HOMA-IR) and the functional capacity of pancreatic β-cells (HOMA-BETA).

(2) $$HOMA\_IR=\frac{\left(\mathrm{Fasting}\;\mathrm{plasma}\;\mathrm{insulin}\;\times \;\mathrm{Fasting}\;\mathrm{blood}\;\mathrm{glucose}\right)}{22.5}$$

(3) $$HOMA\_BETA=\frac{\left(\mathrm{Fasting}\;\mathrm{plasma}\;\mathrm{insulin}\;\times \;20\right)}{\left(\mathrm{Fasting}\;\mathrm{blood}\;\mathrm{glucose}-3.5\right)}$$

### 2.4. Dual Energy x-Ray Absorptiometry (DEXA)

The animals were anesthetized with Sodium Tiopental (Cristália, Itapira, SP, Brazil) at a dose of 0.2 U/g body weight and placed in a dual energy in vivo radiological absorptiometry device (Biocompare In-Vivo Imaging System FX PRO, San Francisco, CA, USA) for the quantification of abdominal adipose tissue reserves. The images captured had the regions of interest (ROI) delimited and their pixels were quantified for analysis using ImageJ software.

### 2.5. Glycogen Measurement 

The liver was collected after the euthanasia of the animals at two different cohorts: after the 24-hour fasting period or after the 24-hour ad libitum food ingestion period, i.e., either the CT and IF groups with free access to food or the CT and IF groups in 24-hour fasting. In total, 250 g of each sample was weighed and placed in a 15-ml conical tube. Then, 1 mL of 30% potassium hydroxide (Sigma-Aldrich, St Louis, MO, USA) was added to the tissues and boiled for 1 hour. After that, 100 µL of saturated sodium sulfate solution (Sigma-Aldrich, St Louis, MO, USA) and 3.5 mL of 70% ethanol (Synth, Diadema, SP, Brazil) were added to the mixture and the samples were boiled for 15 minutes and centrifuged at 3000 RPM for 7 minutes. The supernatant was discarded and the ethanol wash was repeated. The samples were resuspended in 1 mL of hot water and then 50 µL of the sample and 200 µL of 0.2% antrona concentrated sulfuric acid solution (Sigma-Aldrich, St Louis, MO, USA) were placed in a 96-well plate. Next, absorbance at a wavelength of 650 nm was recorded by a plate reader (Biotek Sinergy H1 - Winooski, VT, USA). Glycogen concentrations were calculated using the absorbance values, and the liver mass (grams) of tissue was used to normalize glycogen concentrations.

### 2.6. Oral Glucose Tolerance Test 

In the last week of treatment (12th week), both groups were submitted to a 12-hour overnight fast. Then, blood was collected by tail snip for glucose measurement (time 0) on a blood glucose monitor (FreeStyle Potium Neo, Witney, Oxon, Uk). Later, a glucose solution of 2g/kg per body weight was administered to the animals through gavage, and blood glucose concentrations were measured after 5, 10, 15, 30, 60, 90, and 120 min.

### 2.7. Intraperitoneal Insulin Tolerance Test

In the last week of treatment (12th week), in a cohort different from that in which we performed oGTT, animals were submitted to a 4-hour food restriction. Then, blood was collected by puncture at the animal caudal end for glucose measurement (time 0) on a blood glucose monitor (FreeStyle Potium Neo, Witney, Oxon, UK). After that, a solution of regular human insulin (Humulin, Indianapolis, IN, USA) at a dose of 0.75 U / kg per body weight was administered to the animals by intraperitoneal injection, and blood glucose concentrations were measured after 5, 10, 15, 30, 60, 90, and 120 min.

### 2.8. Blood Analysis 

Blood was collected for insulin concentrations measurement following an Elisa kit protocol (Milipore, Billerica, MA, USA). Hemoglobin A1c (HbA1c) was also assessed by following the protocol of an automated chemistry analyzer by immunoturbidimetry (Labtest, Lagoa Santa, MG, Brazil).

### 2.9. Isolation of Pancreatic Islets

The isolation of pancreatic islets was carried out by the method of exocrine pancreas digestion using collagenase V [54], in which 20 mL of collagenase solution (0.68 mg/mL—Sigma-Aldrich, St. Louis, MO, USA) was injected through the bile duct. The dissected pancreas was placed in a bath at 37 °C for 25 min and shaken by hand for exocrine pancreas digestion. The sample was washed to remove exocrine tissue, and pancreatic islets were collected using a micropipette and a stereoscope. 

### 2.10. Static Insulin Secretion

Pancreatic islets isolated from Wistar rats were placed in a microtube containing Krebs-Henseleit buffer with 0.1% albumin and 5.6 mM glucose for 30 min. Next, the islets were incubated at 37 °C in a microtube containing Krebs-Henseleit with 2.8, 5.6, 8.3, 11.1, or 16.7 mM glucose. Then, the supernatant and sonicated islets were stored at -20 °C for further measurements. The amounts of secreted and intracellular insulin content were determined by radioimmunoassay (RIA) [55].

### 2.11. Pancreas Histological Assessment 

The pancreas was dissected, collected, and fixed with 40 mL of 10% formalin. Then, the pancreases were paraffin-embedded and sectioned at 4μm using a semi-automated microtome (RM2155 Leica Micro-systems, Wetzlar, Hessen, Germany). Afterward, the tissue sections were mounted on glass slides. The sections were stained with hematoxylin and eosin (H&E). All slides were examined using light microscopy with a camera attached (Nikon Eclipse TS100, Sao Paulo, SP, Brazil) under a magnification of X200. The slides were scanned and all pancreatic islets found were photographed. The images were calibrated and analyzed with the Aperio ImageScope software (Leica Micro-systems, Wetzlar, Hessen, Germany), then the length and width of each islet were measured. Next, the area of each pancreatic islets present on each slide was quantified by multiplying length and width.

### 2.12. Cell Viability

For the analysis of cell viability, groups of 20 pancreatic islets were dissociated, and the cells were incubated with ViaCount reagent (Millipore, Billerica, MA, USA) for 5 minutes at room temperature. The samples were placed into a 96-well plate for flow cytometer reading (Guava easyCyte ™ 8Ht Sampling - Millipore, Billerica, MA, USA), and cell viability, cell apoptosis, and cell death were quantified by counting 1000 events.

### 2.13. Measurement of Net ROS Production

Previously isolated islets were pre-incubated with Krebs-Henseleit buffer containing 5.6 mM glucose. Next, the samples were maintained for 1 hour in Krebs-Henseleit buffer containing 2.8 or 16.7 mM glucose. Then, the samples were incubated for 20 min with redox sensitive probe 50 μM dihydroethidium (DHE– Life Technologies, Eugene, Oregon, EUA) or 15 µM MitoSOX Red reagent (Life Technologies, Eugene, Oregon, USA—5 μM). After that, 300 μL of trypsin (Gibco, Grand Island, NY, USA) was added for 2 min to disperse the cells. Afterward, for trypsin inactivation, 600 μL of RPMI-1640 culture medium with 5% fetal bovine serum was added (Life Technologies, Itapevi, SP, Brazil). The islets were recollected and homogenized in 200 μL of RPMI-1640 culture medium for cell dispersion. The samples were placed in a 96-well plate and analyzed by flow cytometry (Guava EasyCyte 8HT- Millipore, Billerica, MA, USA).

### 2.14. Measurement of Net Hydrogen Peroxide Production 

Groups of 120 pancreatic islets were placed in a microtube and pre-incubated for 30 min at 37 °C with in Krebs-Henseleit buffer with 5.6 mM glucose and 0.1% albumin. After this period, the islets were incubated for 1 hour at 37 °C with 2.8 or 16.7 mM glucose. Then, the samples were sonicated and incubated for 30 minutes with 50 μM Amplex Red probe (Life Technologies, Eugene, OR, USA), a specific marker for hydrogen peroxide (H_2_O_2_). The samples were placed into a 96-well plate for absorbance reading at 560 nm in a microplate reader (Biotek, Winooski, VT, USA). 

### 2.15. Western Blot Analysis 

To evaluate protein kinase B phosphorylation (p-AKT), samples of liver, retroperitoneal white adipose tissue (WAT), and extensor digitorum longus muscle were removed before and after an intravenous administration of 10 U regular insulin (Eli Lilly and Company, Indianapolis, IN, USA). To evaluate the expression of mitochondrial superoxide dismutase (SOD2) and glutathione peroxidase 1 (GPX1), groups of 300 pancreatic islets were collected after euthanasia of the animals. The tissues were collected in radioimmunoprecipitation assay buffer (Thermo Scientific, Saint Louis, MI, USA) containing protease and phosphatase inhibitors. The samples were sonicated, laemmli buffer was added, and the samples were boiled for 5 min. After that, polyacrylamide gel electrophoresis was performed followed by transfer to a nitrocellulose membrane. After transfer, the membranes were blocked with 5% bovine albumin solution for one hour at room temperature and incubated overnight at 4 °C with primary antibody (Millipore, Darmstadt, Hesse, Germany). They were incubated with the secondary antibody (Bio-Rad Laboratories Inc., Hercules, CA, USA) for 1 hour at room temperature. Finally, the membranes were developed using the enhanced chemiluminescent (ECL) reagent, and the images were captured by the Image Quant LAS4000 apparatus (GE Healthcare, Uppsala Sweden). The quantitative analysis of the bands was made by densitometry with the program of the Image Quant apparatus. Protein expression was normalized by the expression of the constitutive α-tubulin protein as the control protein or through the stained membranes with Ponceau-S to control protein loading.

### 2.16. Statistical Analysis

The results are expressed as the mean ± standard error of the mean (SEM). GraphPad Prism 5 software was used for analysis. Differences between multiple conditions were determined by One-way ANOVA followed by Bartlett’s test for equal variances or two-way ANOVA followed by Sidak’s multiple comparisons test, as specified in figure legends. In experiments with only two conditions, the differences were determined by Student’s t-test. Comparisons were considered significantly different for *p* < 0.05. 

## 3. Results

### 3.1. Body Weight Gain and Development 

**Figure 2:**
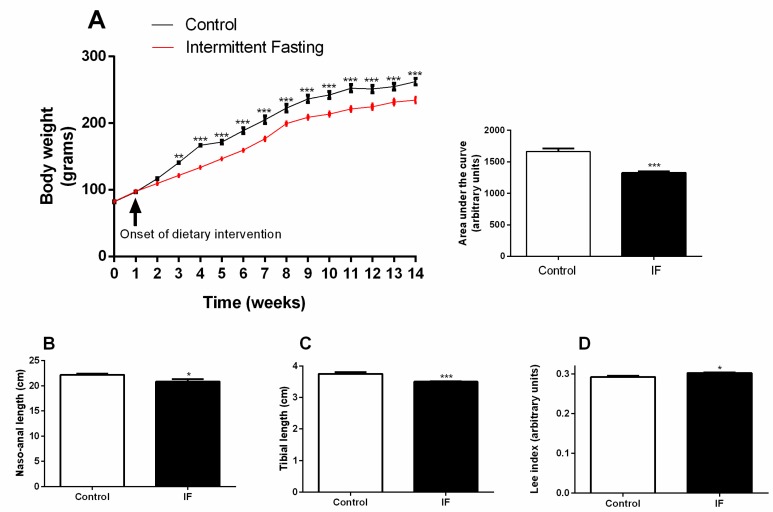
(A) Weekly body weight, (**B**) naso-anal length, (**C**) tibia length, and (**D**) the Lee index of Wistar rats submitted to intermittent fasting (IF) for 12 weeks. The results are presented as the means ± standard error of the mean (SEM) with 10 different animals for each group. * *p* <0.05, ** *p* <0.005, and *** *p* <0.0005 compared to the control of the same period, as indicated by two-way ANOVA followed by Sidak’s multiple comparisons test (**A**) or Student’s t-test (**B**–**D**).

Thirty-day-old Wistar rats were randomly divided into two groups: control (CT) and submitted to intermittent fasting (IF) for 12 weeks. Lower weight gain was recorded in the IF group already after the second week of dietary intervention (Figure 2A—week 3). These changes were maintained throughout the whole treatment period; the area under the curve of the treated animals was 20.3% lower than the control animals (Figure 2A). At the end of the treatment, the tibia length and naso-anal length were significantly decreased in the IF group (Figure 2B,C), and this led to an increased Lee index (Figure 2D). 

### 3.2. Food Intake and Stomach Disturbances

**Figure 3:**
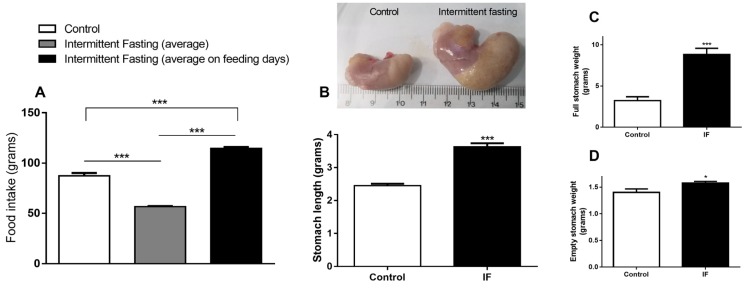
(**A**) Food intake, (**B**) stomach length, and (**C**) full and (**D**) empty stomach weight of Wistar rats submitted to IF for 12 weeks. The results are presented as the means ± standard error of the mean (SEM) with 10 different animals for each group. * *p* <0.05 and *** *p* <0.0005 compared to the control of the same period, as indicated by one-way ANOVA followed by Bartlett’s test for equal variances (A) or Student’s t-test.

The IF group consumes 35% less chow compared to the control group if the average total intake is considered, i.e., fasting days (zero consumption) plus feeding days (gorging behavior). However, if we consider only the mean ingestion of ad libitum ingestion days, the consumption in relation to the control is 31% higher, indicating chow overconsumption (Figure 3A). Figure 3B,C show that this hyperphagia caused a large increase in stomach length (by 47.95%) and weight (by 171.66%). Even after emptying stomach contents, we observed increased stomach weight by 12.55% (Figure 3D).

### 3.3. Body Composition

**Figure 4:**
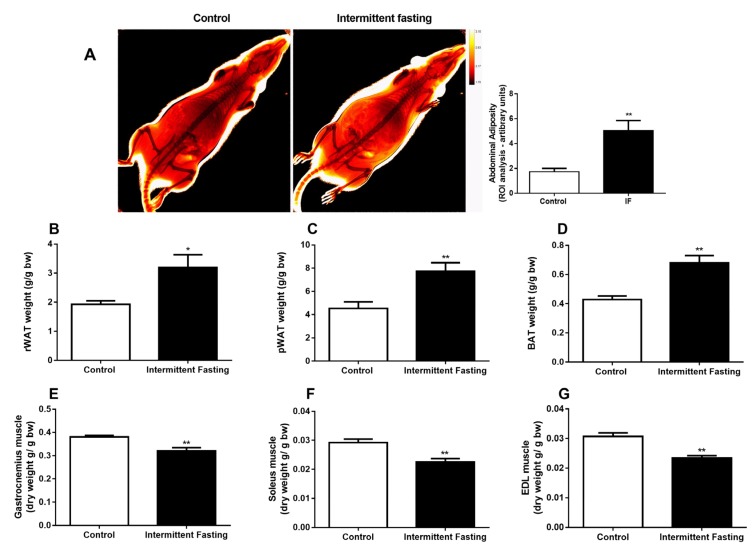
(**A**) Dual energy x-ray absorptiometry (DEXA), (**B**) retroperitoneal, (**C**) perigonadal, and (**D**) brown adipose tissue weight. (**E**) Dry gastrocnemius weight, (**F**) Soleus, and (**G**) Extensor digitorum longus (EDL) muscle of Wistar rats submitted to IF for 12 weeks. The results are presented as the means ± standard error of the mean (SEM) with 10 different animals for each group. * *p* <0.05 and ** *p* <0.005 compared to the control of the same period, as indicated by Student’s t-test.

In vivo dual energy x-ray absorptiometry showed increased abdominal adiposity, as can be seen in Figure 4A. In addition, the weights of adipose tissues (Figure 4B–D) and dry muscles (Figure 4E–G) reveals changes in body composition with fat mass gain and muscle loss in the IF group. 

### 3.4. Liver Alterations

**Figure 5:**
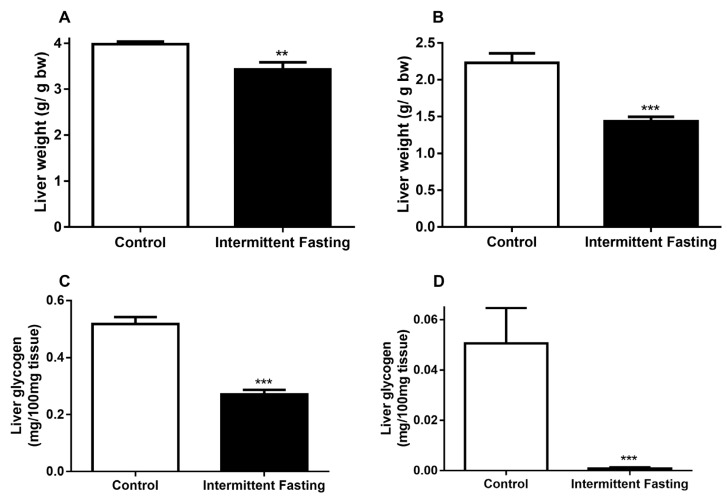
(**A**) Liver weight before and (**B**) after 24h of fasting, (**C**) liver glycogen content before and D) after 24h of fasting of Wistar rats submitted to IF for 12 weeks. The results are presented as the means ± standard error of the mean (SEM) with 10 different animals for each group. ** *p* <0.005 and *** *p* <0.0005 compared to the control of the same period, as indicated by Student’s t-test.

IF reduced liver weight in the fed state by 13.8% (Figure 5A) and after fasting by 35.68% (Figure 5B) when compared to the control in a similar state, whereas the reduction in liver weight may be correlated with reduced glycogen stores. We analyzed glycogen content in both states. In the fed state, a 47.68% reduction in glycogen (Figure 5C) was observed, and fasting led to a 98.33% liver glycogen decrease in the IF group (Figure 5D). 

### 3.5. Glucose Homeostasis 

**Figure 6:**
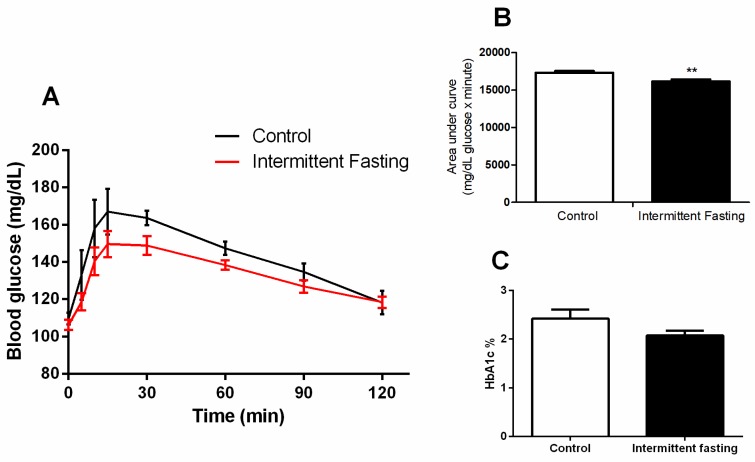
(**A**) Oral glucose tolerance test (oGTT), (**B**) area under curve, and (**C**) hemoglobin A1c (HbA1c) of Wistar rats submitted to IF for 12 weeks. The results are presented as the means ± standard error of the mean (SEM) with 5 different animals for each group. ** *p* <0.005 compared to the control, as indicated by Student’s t-test (B and C), two-way ANOVA followed by Sidak’s multiple comparisons test (A).

An oral glucose tolerance test (oGTT) performed at the end of treatment showed no differences between the control and IF group (Figure 6A). The area under the curve was significantly lower in this group with a reduction of 6.8% (Figure 6B). However, hemoglobin A1c levels after IF treatment do not differ significantly compared to the control group (Figure 6C). 

**Figure 7:**
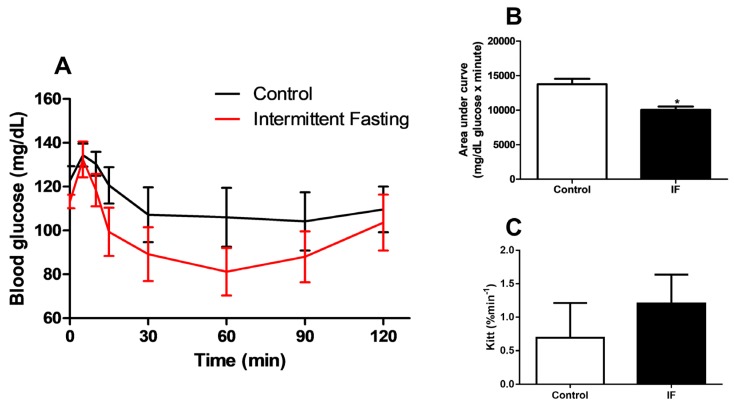
(**A**) Intraperitoneal insulin tolerance test (iITT), (**B**) area under curve and (**C**) glucose decay constant rate during insulin tolerance test (kITT) of Wistar rats submitted to IF for 12 weeks. The results are presented as means ± standard error of the mean (SEM) with 5 different animals for each group. * *p* <0.05 compared to the control, as indicated by Student’s t-test (B and C), two-way ANOVA followed by Sidak’s multiple comparisons test (A).

At the end of the treatment, an intraperitoneal insulin tolerance test (iITT) was also performed. Although the area under the curve was 27% lower in the IF group (Figure 7B), the groups did not show significant differences in blood glucose values at any of the times studied (Figure 7A) and the glucose decay constant (Kitt) did not differ between groups (Figure 7C).

### 3.6. Insulin Concentrations

**Figure 8:**
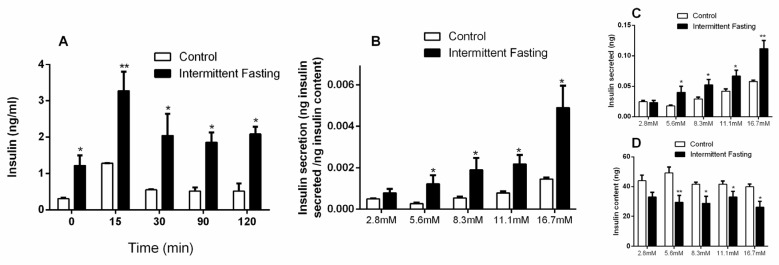
(**A**) Insulin concentrations obtained during oral glucose tolerance tests (oGTT) in Wistar rats submitted to IF for 12 weeks. (**B**) Content-corrected insulin secretion, (**C**) insulin secretion, and (**D**) insulin content values in pancreatic islets isolated from Wistar rats submitted to IF for 12 weeks, after one hour of incubation in the presence of 2.8, 5.6, 8.3, 11.1, and 16.7 mM glucose. The results are presented as the means ± standard error of the mean (SEM) with 5 different animals for each group. * *p* <0.05 and ** *p* <0.005 compared to the control at the same time, as indicated by two-way ANOVA followed by Sidak’s multiple comparisons test.

Plasma insulin concentrations were measured in the fasted state and after 15, 30, 90, and 120 min of oral glucose administration. Intermittent fasting for 12 weeks greatly increased basal plasma insulin concentrations (by 4-fold). Insulin increase was also observed in the IF group by 155%, 271%, 259.7%, and 304% after 15, 30, 90, and 120 min of oral glucose stimulation (Figure 8A), respectively.

A glucose-stimulated insulin secretion (GSIS) assay was performed with isolated pancreatic islets after glucose stimulation at 2.8, 5.6, 8.3, 11.1, and 16.7 mM levels. Content-corrected GSIS and secreted insulin (Figure 8B,C) were significantly higher after intermittent fasting at all glucose concentrations, except in presence of 2.8 mM glucose. At the same time, the insulin content remaining in the pancreatic islets of the IF group was lower at all glucose concentrations, with the exception of low-level glucose (Figure 8D), which corroborates the higher values of insulin found.

### 3.7. Homeostasis Model Assessment (HOMA) Indexes 

**Figure 9:**
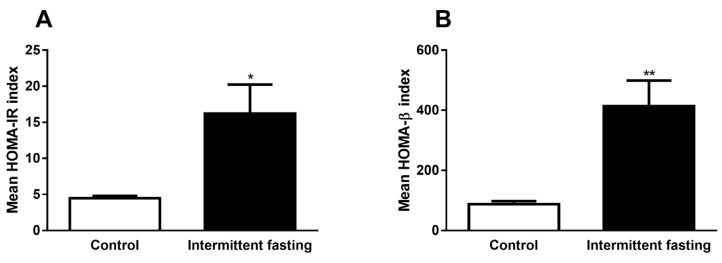
(**A**) Homeostasis model assessment of insulin resistance (HOMA-IR) and (**B**) homeostasis model assessment of β-cell function (HOMA-β) indexes of Wistar rats submitted to IF for 12 weeks. The results are presented as the mean ± standard error of the means (SEM) with 5 different animals for each group. * *p* <0.05 and ** *p* <0.005 compared to the control, as indicated by Student’s t-test.

From the values of fasting blood glucose and fasting plasma insulin, both obtained during oGTT performed in the last week of dietary intervention (time 0 – before glucose administration), we calculated HOMA-IR and HOMA-β indexes, which are mathematical models used to evaluate insulin resistance. Intermittent fasting greatly increased values in both models by 3.6-fold (HOMA-IR) and by 4.7-fold (HOMA-β), as can be observed in Figure 9A,B.

### 3.8. AKT Phosphorylation 

**Figure 10:**
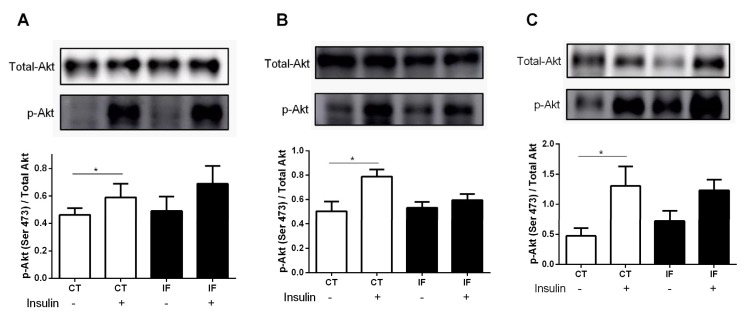
(**A**) Protein kinase B phosphorylation (p-AKT) expression of extensor digitorum longus (EDL) muscle, (**B**) liver, and (**C**) retroperitoneal white adipose tissue from Wistar rats submitted to IF for 12 weeks. The results are presented as the means ± standard error of the mean (SEM) with 5 different preparations for each group. * *p* <0.05 compared to the respective control, as indicated by Student’s t-test.

Figure 10 shows that 12 weeks of intermittent fasting were able to alter AKT phosphorylation in muscle, liver, and white adipose tissue (WAT). In the control group, an increase in AKT phosphorylation after intravenous insulin stimulation was observed, while the IF group did not show significant differences in AKT phosphorylation, indicating impairment in insulin action (Figure 10A–C).

### 3.9. Pancreatic Islet Area and Viability of Cells from Pancreatic Islets

**Figure 11:**
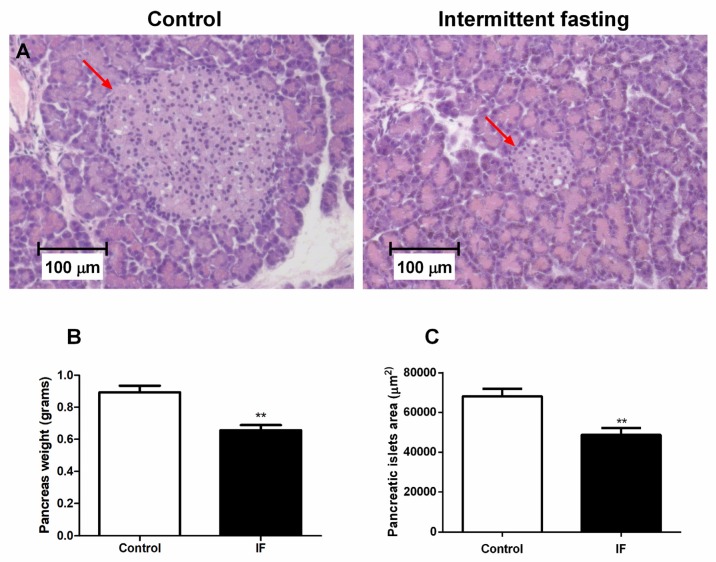
(**A**) Pancreatic islet of the longitudinal cut of pancreas caudal portion, (**B**) pancreas weight related to body weight, and (**C**) pancreatic islets area from Wistar rats submitted to IF for 12 weeks (Hematoxylin-eosin stained tissue (H&E) and 100x magnification). The results were presented as the mean ± standard error of the mean (SEM) with 5 different animals for each group. ** *p* <0.005 compared to the control, as indicated by Student’s t-test.

Histological analyzes in pancreas revealed a 28.6% decrease in the islet size of the animals submitted to IF treatment (Figure 11A,C). The weight of the pancreas also showed a significant reduction in the IF group of about 26% (Figure 11B).

**Figure 12:**
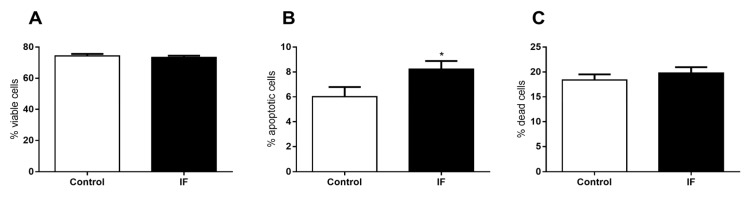
(**A**) Viability, (**B**) the percentage of apoptotic cells, and (**C**) the percentage of dead cells of dispersed cells from pancreatic islet isolated from Wistar rats submitted to IF for 12 weeks. The results are presented as the mean ± standard error of the mean (SEM) with 5 different cellular preparations for each group. * *p* <0.05 compared to the control, as indicated by Student’s t-test.

The viability of the dispersed cells from pancreatic islets was assessed and no significant difference in the number of viable cells (Figure 12A) and number of dead cells was observed (Figure 12C). However, we observed a significant increase of 27.8% in the number of apoptotic cells (Figure 11B).

### 3.10. ROS Production of Dispersed Cells from Pancreatic Islets 

**Figure 13:**
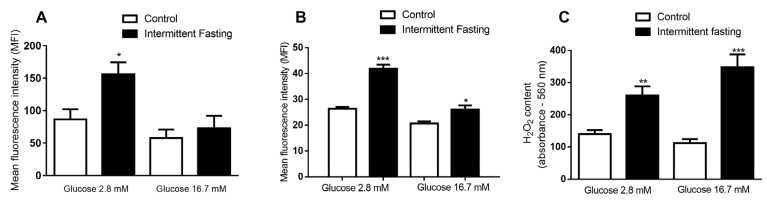
(**A**) Mean fluorescence intensity (MFI) emitted by a dihydroethidium (DHE) probe, (**B**) MFI emitted by a MitoSox Red probe, and (**C**) the absorbance emitted by the Amplex Red probe of dispersed cells from a pancreatic islet isolated from Wistar rats submitted to IF for 12 weeks after one hour of incubation in the presence of 2.8 and 16.7 mM glucose. The results are presented as the means ± standard error of the mean (EPM) with 6 different cellular preparations for each group. * *p* <0.05, ** *p* <0.005, and *** *p* <0.0005, compared to the respective control, as indicated by Student’s t-test.

We observed an 80.2% increase in the net fluorescence induced by reactive oxygen species (ROS) in the presence of glucose 2.8 mM after incubation with the redox-sensitive probe DHE (Dihydroethidium). No significant difference was found between the groups at 16.7 mM glucose level (Figure 13A).

Net mitochondrial ROS production was also measured in the presence of 2.8 and 16.7 mM glucose after incubation with the MitoSox Red probe (Figure 13B). After 12 weeks of intermittent fasting, a significant increase in fluorescence associated to ROS production was revealed (59.2% in low glucose and 26% in high glucose concentration).

Finally, we measured the concentrations of hydrogen peroxide (H_2_O_2_) in the presence of 2.8 and 16.7 mM of glucose after incubation with the Amplex Red probe (Figure 13C). IF caused a significant increase in H_2_O_2_ detection in the presence of both glucose concentrations compared to the values of the control group. At low glucose levels, an increment of 85% was observed, while, at at 16.7 mM glucose, an increase of 211% was measured.

### 3.11. Antioxidant Systems 

**Figure 14:**
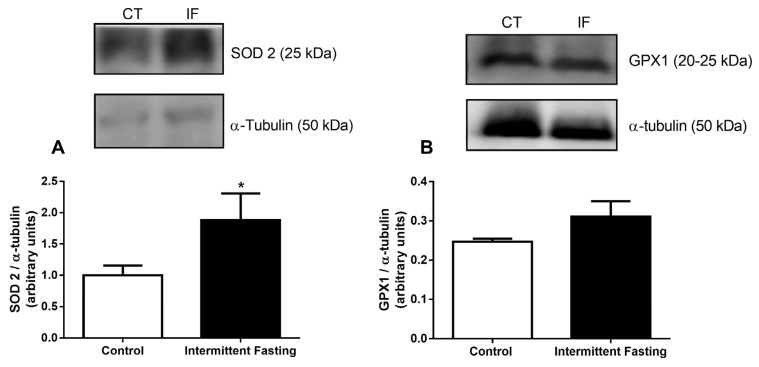
(**A**) Superoxide dismutase 2 (SOD2) and (**B**) glutathione peroxidase 1 (GPX1) expression of pancreatic islets isolated from Wistar rats submitted to IF for 12 weeks. The results are presented as the means ± standard error of the mean (SEM) with 5 different cellular preparations for each group. * *p* <0.05 compared to the control, as indicated by Student’s t-test.

Lastly, we analyzed two antioxidant enzyme expressions: mitochondrial superoxide dismutase (SOD2), also known as manganese-dependent superoxide dismutase (MnSOD), and glutathione peroxidase 1 (GPX1). The enzymes were measured by electrophoresis gel and calculated in relation to α-tubulin protein expression. Intermittent fasting resulted in a significant increase (by 70.86%) in SOD2 expression (Figure 14A). However, after intermittent fasting, GPX1 expression was not different from the values found in the control group. 

## 4. Discussion 

Alternate-day intermittent fasting (IF) is a relatively new dietary approach that has been promoted to help weight management. As a result, the number of people adhering to this new diet grows every day. We are aware that children are not usually placed on IF diets. However, our society is constantly changing, and with increasing levels of obesity and overweight in children, it is possible that this may be a dietary approach recommended by nutrition professionals in the future. Hence, we need to study possible consequences of this practice in young organisms, as the adverse effects of alternate-day IF have not been fully elucidated.

Compared with daily calorie restriction, intermittent fasting in obese volunteers did not produce better adherence, weight loss, weight maintenance, or improvement in risk indicators for cardiovascular disease. Rather, the dropout rate and hunger in the IF fasting group was higher than that in the daily calorie restriction group, and the authors concluded that IF may be less sustainable in the long-term for obese individuals [56,57]. 

In this study, long-term IF was successful in reducing body weight gain (Figure 2A) in female Wistar rats, and this may be due to lower average food intake (by 35%) (Figure 3A). Weight loss as a consequence of IF has been reported in previous studies in both animal [58,59] and human subjects [60,61,62]. However, Sakamoto and Grunewald showed that IF in 4-week-old rats markedly reduced the growth rate and the animals had smaller livers, kidneys, hearts, tibias and tibialis anterior muscles [63]. In this study, the treatment was also initiated in young animals (4-weeks old) and, as can be seen in Figure 2B (reduced naso-anal length) and 2C (reduced tibial length), may affect growth since the animal is developing [64]. 

As previously reported, IF in slim and healthy people for 2 weeks [65] or 1 month [66] showed a significant decrease in resting energy expenditure. Hence, the animals could gain weight in relation to control animals if the food intake in both groups were the same, assuming that IF promotes lower energy expenditure. Additionally, a hyperphagic behavior on free feeding days (Figure 3A) was observed, which caused a large increase in stomach dimensions (3B-D). There are records of IF-induced increased expression of the agouti-related peptide (AGRP), neuropeptide Y (NPY) and orexin due to intermittent fasting in rodents, even when the stomach was full [58,67,68]. These orexigenic neurotransmitters are involved in appetite modulation and metabolic regulation [69], and their increasing concentrations in plasma could explain the increase in food intake. There are also several human studies showing increased subjective appetite sensation as a result of fasting cycles [70,71,72].

It has been previously reported that IF promotes a greater deposition of triacylglycerides in the white adipose tissue by increasing the expression of genes involved in lipid storage, such as fatty-specific protein 27 (FSP27) [73]. In 1928, Lee described a rapid method to quantify obesity from body weight and naso-anal length values. The result defines the nutritional status, called the Lee index, which correlates positively with adipose tissue mass. [74,75]. In this study, the values obtained from this index reveal a slight, but significant, increase in the IF group values compared to the control group (Figure 2D), which does not mean that the IF animals are obese, but corroborates the largest adipose reserve found (Figure 4A–D).

No significant differences were found between groups neither in blood glucose concentrations after oral glucose administration (Figure 6A) nor in HbA1c (Figure 6C). These results contradict previously published studies, which show a reduction in blood glucose concentrations. [25,76,77]. Nevertheless, in this case, the lower glycaemia can be a consequence of large amounts of circulating insulin concentrations (Figure 8) since insulin is the hypoglycemic hormone that allows glucose to enter in insulin-sensitive target cells. The decrease in liver weight observed may be related to lower glycogen reserves (Figure 5). The significant drop in hepatic glycogen and muscle glycogen concentrations (Supplemental Figure S2) may result in increased fatigue and impaired maintenance of normoglycemia between feeding periods [78]. 

Our data show changes in body composition with decreased muscle mass (Figure 4E–G) and increased fat mass (Figure 4B–D), in contrast to the literature data, which generally show reduced body fat and maintenance of lean mass. [28,62,79,80,81]. However, a decrease in lean mass after only 1 month of IF has been reported in adolescent girls [42]. An increase in body mass index was found with IF only in women, with a reduction in this parameter in men [82]. Another study found that improvements in body composition are less pronounced in younger women [62]. In female mice, IF did not cause weight loss [83]. It is possible that intermittent fasting causes more adverse effects on the body composition of females, and this could be accentuated in younger and developing organisms.

Glucose and insulin plasma concentrations under fasting conditions in both healthy and type 2 diabetes mellitus (T2DM) subjects are expressed at characteristic concentrations for each individual’s nutritional status. Basal insulin concentrations are a consequence of fasting glucose concentrations, the secretory capacity of pancreatic β-cells, and the rate of pulsatile insulin secretion. Previous work shows that IF increases both basal and glucose-stimulated insulin secretion in mice with diet-induced obesity [83]. This increase in insulin concentrations (Figure 8A), also found in in vitro pancreatic islets (Figure 8B–D), suggests resistance to this hormone, as evidenced by a significant increase in the homeostasis model assessment (HOMA) index (Figure 9), and impaired AKT phosphorylation in muscle, liver, and adipose tissue (Figure 10). 

HOMA is a mathematical calculation based on fasting insulinemia and glycaemia, proposed by David Matheus [53] as a simple and fast way to determine insulin resistance (HOMA-IR) and the functional capacity of pancreatic β-cells (HOMA-BETA). Similar data were found in a study performed with male rats submitted to 5 weeks of IF and likewise in volunteers during 1 month of fasting, both showing increased plasma insulin and HOMA indexes [38,84]. 

One hypothesis for this insulin resistance induced by fasting is increased secretion of ghrelin. The secretion of this gastric hormone is elevated in calorie restricted mice, rats, and humans [85]. Ghrelin is produced and secreted predominantly in the oxyntic mucosa of stomach (approximately 60–70% of circulating ghrelin), and low plasma ghrelin concentrations are associated with elevated fasting insulin concentrations and insulin resistance, suggesting both physiological and pathophysiological roles in glucose metabolism [86,87]. Barazzoni et al. observed that, in rats, sustained ghrelin administration reduced hepatic AKT phosphorylation. The deregulation of AKT phosphorylation has been suggested to occur under insulin resistance conditions and diabetes [88].

Although insulin tolerance tests showed no differences in glycaemia values at any time studied (Figure 7A), nor in glucose decay constant values (Figure 7C), impaired AKT phosphorylation in muscle, liver, and WAT indicates impaired insulin action (Figure 10). Intermittent fasting for 8 months in rats caused impaired glucose clearance, as reported by Cerqueira et al. [89]. In addition, intermittent food restriction in female rats for 6 weeks induced glucose intolerance and detrimental hypothalamic alterations coupled with compulsive eating behavior [90]. Thus, frequent feeding and fasting cycles may be harmful and associated with insulin resistance, increasing risks of T2DM. A hyperinsulinemic-euglycemic clamp was not conducted in this work and should be included in future studies.

There are several studies that suggest that high insulin concentrations are associated with typical pathologies of the metabolic syndrome, such as insulin resistance [91,92,93,94,95,96,97], obesity [98,99,100,101,102,103], hypertension, cardiovascular diseases [104,105,106,107,108], atherosclerosis [109], and hepatic steatosis [110,111]. In general, the development of T2DM begins when the metabolic demand for insulin is greater, due to peripheral insulin resistance. This insulin resistance generally precedes the development of hyperglycaemia. In other words, there is a period of normoglycaemia, where pancreatic β-cells compensate insulin resistance by increasing insulin secretion [112,113,114,115]. However, this compensatory hyperinsulinemia can cause damage to the secretory cell in the long run, leading to β-cell apoptosis and the development of T2DM [116,117,118]. 

Corroborating with this idea, the decreased pancreas weight and pancreatic islet area (Figure 11), as well as increased apoptotic cells in pancreatic islets (Figure 12), reinforce the hypothesis that IF may be detrimental to the endocrine pancreas and represents an unhealthy long-term diet. Although we must be cautious when comparing the effects to humans, this study in an animal model is an important tool to evaluate the potential impacts of this diet in a standardized manner, with minimal artifact interference. Moreover, animal studies allow us to evaluate the effects directly in important organs, such as the pancreas, given that data in humans is obviously sparse or non-existent as to whether long-term intermittent fasting diets affect pancreatic islets. 

Lastly, 8 weeks of IF caused no difference in net ROS production between groups at any of the glucose concentrations studied (Supplemental Figure S3). However, 12 weeks of IF increased total (Figure 13A) and mitochondrial (Figure 13B) net ROS production in the pancreas, besides increasing hydrogen peroxide levels (Figure 13C), together showing that the effects are accentuated by a longer time of treatment and lead to oxidative imbalance, as previously shown by Cerqueira et al. [89] in other tissues. Intermittent fasting in mice showed that, on ad libitum feeding days, when the animal ingests a large amount of food, ROS production is enhanced due to increased mitochondrial respiration [21]. 

These ROS increments caused the enhancement antioxidant defenses, as evidenced by increased SOD2 (Figure 14A), that catalyzes the dismutation of superoxide into O_2_ and H_2_O_2_, which are less damaging molecules. This cellular response promoted by 12 weeks of IF diet may be important to mitigate oxidative stress, since increased ROS production has been reported to cause a loss of function and even apoptosis in several cell types [119,120,121,122,123,124,125,126], including pancreatic islets [127,128,129,130,131,132,133].

The insulin receptor is essential for pancreatic β-cell function and survival. The insulin signaling networks contain many proteins that are established regulators of apoptosis and proliferation. Insulin directly prevents apoptosis in human and mouse islets, as well as in β-cell lines [134,135]. Cerqueira et al. showed that long-term intermittent feeding leads to redox imbalance and peripheral insulin receptor nitration [89]. Thus, increased oxidative stress induced by 12 weeks IF may be accompanied by the oxidation of the insulin receptor in pancreatic β-cells and the impairment of cell survival. However, a more detailed study of oxidative stress induced by fasting on the protective effects of autocrine insulin signaling is needed.

Several studies show that the effects of IF depend on several factors, such as the period of day in which fasting is practiced. Time restricted feeding (TRF) is a type of IF that involves eating all nutrients within a few hours every day, usually up to 12 hours. Studies suggest that, depending on the time of the eating window, TRF leads to opposite effects. Restricting food intake to the resting phase worsened fasting and postprandial glucose concentrations, blood pressure, and lipid concentrations in humans [32,136], and induced leptin resistance that contributes to the development of obesity and metabolic disorders in mice [137]. However, restricting food intake to the active phase improved insulin sensitivity, blood pressure, glucose tolerance, and oxidative stress [8,138,139]. 

Corroborating these results, there are several long-term studies in humans showing detrimental effects of skipping breakfast, which is a type of IF by prolonging overnight fasting to the active phase. Breakfast skipping is associated with a significantly increased risk of overweight and obesity, poorer glycemic control, insulin resistance, and an increased risk of T2DM [140,141,142,143,144,145]. Considering that the present study evaluated juvenile rats and that they present a different endocrinology response to adults, our results only apply to juveniles. In developing organisms, growth hormone may play an important role in inducing insulin resistance under fasting stress conditions, which may be significant for the defense against hypoglycemia [146]. Besides, further long-term research is necessary to investigate which IF protocol is more suitable to reduce these side effects and improve health, before IF can be considered a good alternative for weight management in young individuals.

## 5. Conclusions

Intermittent fasting is effective for weight loss, but the long-term safety has been questioned. Considering increasing levels of obesity and overweight in children, we studied possible consequences of this practice in young animals. We have shown here that 12 weeks of alternate-day intermittent fasting in young female Wistar rats causes several changes that can be detrimental in the long run to young individuals, including the elevation of pancreatic islet cells apoptosis and ROS production. We also found a reduction in pancreatic islet mass, a large increase in insulin secretion, and signs of insulin resistance by reduced AKT phosphorylation in muscle and adipose tissue, which may be a risk factor for T2DM. Besides that, we noticed a remodeling of body composition, with increased body fat and decreased muscle mass. Taken together, these findings suggest that caution may be warranted when unrestrictedly recommending intermittent fasting to young individuals, especially for people with compromised glucose metabolism. This study was conducted in healthy juvenile rats and these findings may not translate into adult humans. Further studies are required.

## Supplementary Materials

The following are available online at https://www.mdpi.com/2072-6643/12/4/1029/s1. Figure S1 Macro-nutrients of the standard rodent chow (Nuvilab, Sao Paulo, SP, Brazil), Figure S2 Gastrocnemius glycogen content, Figure S3 Mean fluorescence intensity (MFI) emitted by dihydroethidium (DHE) probe of dispersed cells from pancreatic islet isolated from Wistar rats submitted to IF for 8 weeks after one hour of incubation in the presence of 2.8 and 16.7 mM glucose.
