# Supplementary figures and images for: Intermittent Fasting for Twelve Weeks Leads to Increases in Fat Mass and Hyperinsulinemia in Young Female Wistar Rats
Source: Nutrients. 2020 Apr 9;12(4):1029. doi: 10.3390/nu12041029 (PMC7230500; doi:10.3390/nu12041029)

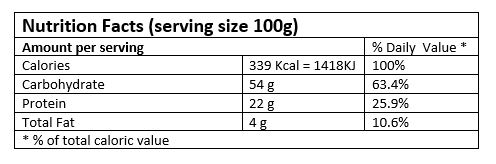

Supplement: Supplementary file 1 [file nutrients-12-01029-s001.zip › supplemental figure 1.JPG]
